# Zinc Removal from the Aqueous Solutions by the Chemically Modified Biosorbents

**DOI:** 10.1007/s11270-017-3661-5

**Published:** 2017-12-16

**Authors:** Krzysztof Rajczykowski, Oktawia Sałasińska, Krzysztof Loska

**Affiliations:** Faculty of Energy and Environmental Engineering, Institute of Water and Wastewater Engineering, Silesian University of Technology, Konarskiego 18, 44-100 Gliwice, Poland

**Keywords:** Biosorption, Zinc, Straw, Chemical modification

## Abstract

Biosorbents are the natural origin adsorbents, which popularity in environmental engineering is steadily increasing due to their low price, ease of acquisition, and lack of the toxic properties. Presented research aimed to analyze the possibility of chemical modification of the straw, which is a characteristic waste in the Polish agriculture, to improve its biosorption properties with respect to removal of selected metals from aquatic solutions. Biosorbents used during the tests was a barley straw that was shredded to a size in the range of 0.2–1.0 mm. The biosorption process was performed for aqueous solutions of zinc at a pH 5. Two different modifications of straw were analyzed: esterification with methanol and modification using the citric acid at elevated temperature. The results, obtained during the research, show a clear improvement in sorption capacity of the straw modified by the citric acid. In the case of straw modified with methanol, it has been shown that the effectiveness of zinc biosorption process was even a twice lower with respect to the unmodified straw. Moreover, it was concluded that the removal of analyzed metals was based mainly on the ion-exchange adsorption mechanism by releasing a calcium and magnesium ions from the straw surface to the solution.

**Graphical Abstract:**
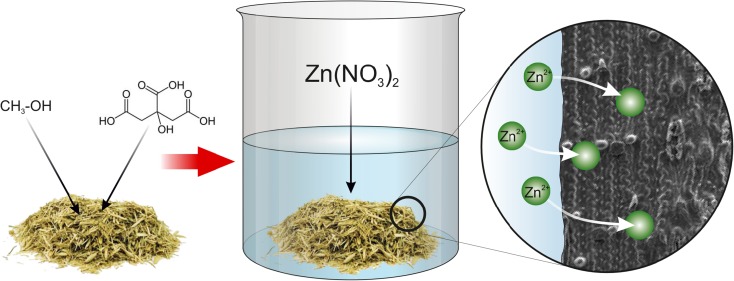
ᅟ

ᅟ

## State of Art

The dynamic development of civilization, which caused the improvement of the quality of life in well-developed countries, on the other hand, contributes to the growth of the environmental pollutions. Still increasing emissions from various industries are becoming a very important problem, forcing to take appropriate action to protect the environment (Mok et al. 2014; Li 2006). One of the significant environmental health hazards is the pollution of surface waters by metals, particularly by heavy metals such as zinc. These metals are getting into the aquatic environment, mostly along with industrial wastewaters (Fang et al. 2018; Singh and Kumar 2017; Kadirvelu et al. 2001). Heavy metal compounds pose a significant threat to the health and life of humans and animals, especially due to the fact that they have the ability to accumulate in organisms, which makes the effects of exposure can occur a long time after exposure time (Jaishankar et al. 2014; Palminger Hallen et al. 1995). Therefore, one of the priorities in the fight against environmental health hazards is to minimize the amount of metal brought to the environment with the industrial wastewaters. Nowadays, there are different methods to reduce the amount of the toxic compounds in industrial wastewater; they can be for example precipitated from aqueous solutions through a chemical precipitation process; however, it usually causes a need for the introduction to the treated wastewaters others, often harmful chemical substances (Farooq et al. 2011).

An alternative for the chemical methods are biotechnological processes which are technologies using a living organisms or residues of living organisms to reduce the contamination level in water without a need of using toxic chemical compounds (Priac et al. 2017; González Vázquez et al. 2016). An example of such biotechnological method is biosorption processes which are the methods for removing selected contaminants using natural, non-toxic sorbents. Biosorption is the phenomenon of removed, for example the molecules of heavy metals, by adsorption on the surface of specially prepared, natural origin sorbent. Another important advantage of biosorption is the possibility of using very cheap and easy to obtain materials as sorbents, such as wastes from the various branches of human life. It is possible to use the biomass generated by plants, animals, and microorganisms (Deniz and Karabulut 2017; Hlihor et al. 2017; Fomina and Gadd 2014). Because of the low-cost and non-toxic materials, biosorption process is increasingly used in environmental engineering.

Numerous studies on the improvement of the biosorbent properties showed that carrying out an appropriate chemical modification of biosorbents can significantly improve the efficiency of impurity removal (Okoli et al. 2016; Deniz and Karabulut 2017; Demirbas 2008; Sciban et al. 2008; Wang and Chen 2009). Effect of modifications on biosorption properties may be the result of developing of the biosorbent contact surface, by increasing its porosity and removing initially adsorbed compounds. Additionally, modification of biosorbents can cause changes in the functional groups located directly on its surface, responsible for binding contaminants (Demirbas 2008).

## Materials and Methods

A straw used during the study was a barley straw, coming from the suburban areas. Before the process of biosorption, straw was washed with distilled water and then dried at the temperature of 60 °C, up to the constant weight. Next, a dry straw was sieved on analytical sieves for selecting further fractions to experiment, with a size range of 0.2–1 mm. After that, a suitable amount of the straw was selected to chemical modifications, leaving some portion as a control sample for comparison purposes. Two types of chemical modifications were used in this study; first one was a citric acid modification and the second was a modification of straw by using the methanol.

A citric acid straw modification was based on the method used by Zhu et al. (2008). In this method, crude straw was treated by the citric acid solution at a high temperature. A significant change relative to the originally used method was an increase of the initial citric acid concentration from 0.6 up to 1 mol/dm^3^ and an increase of the mixing time from 30 min to 4 h. Moreover, the stage of 24 h of mixture incubation was omitted. Straw after the assumed mixing time was transferred to the dryer heated to 120 °C for 90 min. That was a crucial stage of thermochemical modification of the straw structure. After cooling, modified straw was washed with distilled water in an amount of 200 ml per 1 g of straw. Applied changes from baseline methodology allowed to a significant reduction of the modification time. Moreover, a higher citric acid concentration had provided to satisfactory results of improving the biosorption properties of straw. After modification, straw was alkalized by adding a 0.2-mol solution of sodium hydroxide.

The second modification was carried out by flooding a straw by a 99.9% pure methanol in a ratio of 70 cm^3^ of methanol per gram of straw and an additional portion of 0.6 cm^3^ of 0.1 M HCl. Then an obtained mixture of straw was boiled at 60 °C for 48 h, washed with distilled water, and then dried at 60 °C. This method was based on the methodology elaborated by Tiemann et al. (2002), for an alfalfa biomass modification to the lead adsorption processes (Tiemann et al. 2002). Of course, methanol used for modifications is also toxic; thus, as a result of the modification, additional hazardous wastes will be generated. However, unlike heavy metals, that cannot be decomposed at all, but can only change their occurrence form and concentration in particular phases, methanol can be effectively decomposed in many chemical and biotechnological processes. A good example of such processes may be the anaerobic conversion of methanol to methane, which then can be used as a biogas component (Lin et al. 2008). Moreover, there are many environmental engineering processes, including wastewater treatment processes, such as anaerobic biodegradation or a nitrogen removal, where an additive of even contaminated methanol can effectively improve process efficiency (Hwang et al. 2016; Wang et al. 2010). In conclusion, there are many potential possibilities of using methanol-rich wastewaters to improve the efficiency of the selected wastewater treatment processes and that is why it seems reasonable to analyze a possibility of that kind of biosorbent modification.

Biosorption process was carried out in a glass, batch reactors in which the straw, after pouring the zinc solution, was mixed using a magnetic stirrer. All results reported in this paper relate to the average values obtained for a triple repetition of each measurement. The zinc concentration before and after the process was analyzed by using an atomic absorption spectroscopy (AAS), on the apparatus SpectrAA 880 by Varian. In order to accurately determine the biosorption abilities of analyzed straw before and after the modifications, two basic mathematical models of adsorption were used. The first one was a Langmuir adsorption model, which involves the formation of an adsorbate monolayer on the surface of a heterogeneous adsorbent. Linearized form of the Langmuir isotherm which was used during the study allows for an easy and quick comparison of different adsorbents and it takes the following form (Syers et al. 1973):

1 $$ \frac{C_{\mathrm{e}}}{q_{\mathrm{e}}}=\frac{C_{\mathrm{e}}}{q_{\mathrm{max}}}+\frac{1}{b\times {C}_{\mathrm{e}}} $$

where

*q*_e_: The amount of substances adsorbed on the surface of the adsorbent at the adsorption equilibrium (mg/g)
*C*_e_: Concentration of heavy metals remaining in the solution in the adsorption equilibrium (mg/dm^3^)
*q*_max_: Maximum adsorption capacity of the adsorbent (mg/g)
*b*: the Langmuir isotherm constant.

The second adsorption isotherm was a Freundlich isotherm which is an example of typical empirical adsorption model. This type of adsorption isotherm often better fits the results than a Langmuir isotherm, especially in the case of high-energy heterogenic surface of the adsorbents. During the study, a following linearized form of the Freundlich isotherm was used (Sposito 1980):

2 $$ \log \left({q}_{\mathrm{e}}\right)=\frac{1}{n}\log \left({C}_{\mathrm{e}}\right)+\mathit{\log}\left({K}_{\mathrm{f}}\right) $$

where

*K*: a Freundlich constant (often called a Freundlich capacitive coefficient)
*n*: the Freundlich exponent coefficient, defining a degree of surface energy heterogeneity of the adsorbent.

Evaluation of the fitting between proposed models and obtained experimental results was determined by comparing of the *R* ^2^ determination coefficients for linearized form of adsorption isotherms. The final stage of the study was a statistical analysis of the obtained results obtained to confirm their statistical significance. Statistical analysis was made by using a one-way ANOVA analysis, which allows to a verification of the significance of the results by comparing a calculated *F* statistics for each of the analyzed systems with the critical values of *F* statistics, contained in the Fisher-Snedecor distribution tables. All calculations required for statistical analysis were made by using a Statistica software by StatSoft.

## Results and Discussion

**Fig. 1:**
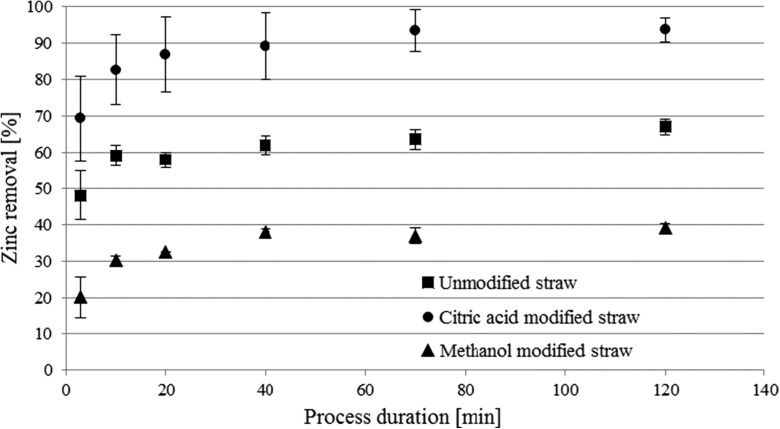
Comparison of the zinc removal degree at the different time during the biosorption processes using a modified and unmodified straw

The conducted preliminary studies revealed that the best pH for the zinc adsorption process is equal 5. In that pH, zinc removal reaction is highly effective, but the solution is clear, without any turbidity and opalescent effects, which could indicate that the metal particles started to fall out of solution because of too high pH value. The first stage of the study was to determine a duration of the adsorption process until it reached a steady state, with no further changes of zinc concentration in the solution. The results of zinc concentration changes during the time of the adsorption process were shown at the figure below (Fig. 1).

**Fig. 2:**
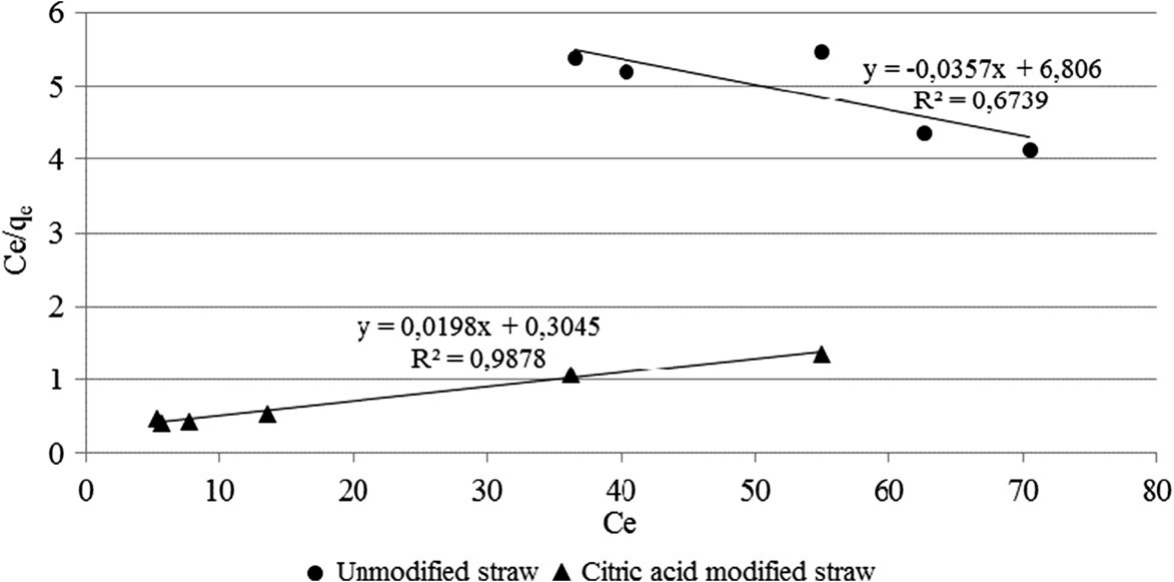
Linearized form of the Langmuir adsorption isotherms for straw modified by citric acid and unmodified

On the basis of presented results, it was assumed that time needed to achieve a steady state of the biosorption process is about 60 min. Next step of the study was to carry out a biosorption process at different mass to volume ratio between straw mass and zinc solution volume, in order to obtain the data necessary to determine respective adsorption isotherms, allowing for more accurate analysis of different biosorbent modification methods. In the case of straw modified with methanol, the degree of zinc removal from the solution was too low to allow a proper determination of the adsorption isotherms, due to the fact that the average value of zinc removal was comparable with a standard deviation for these samples. Therefore, at the figure below, it is only a comparison of adsorption isotherms for the unmodified straw and modified with citric acid (Fig. 2).

**Fig. 3:**
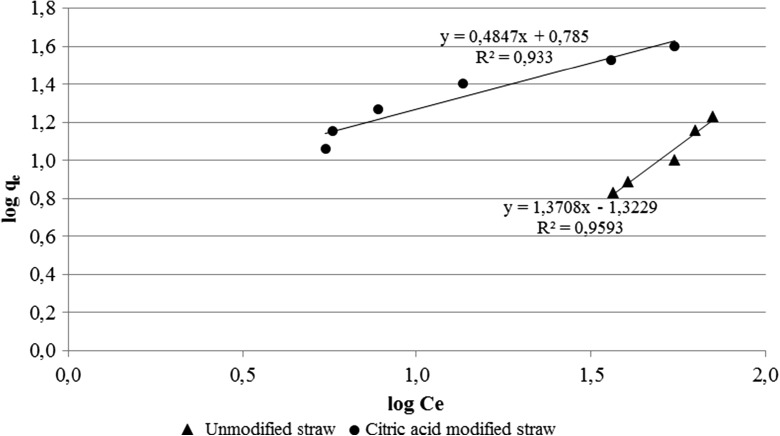
Linearized form of the Freundlich adsorption isotherms for straw modified by citric acid and unmodified

Analysis of the Langmuir adsorption isotherm plot revealed that in the case of straw, unmodified adsorption process was proceeded contrary to the Langmuir model assumptions and therefore it is impossible to compare both types of biosorbents based on the parameters determined by this type of adsorption isotherm. That is why results were compared based on the assumptions of the Freundlich isotherm model, which was presented at the figure below (Fig. 3).

In the case of the Freundlich adsorption isotherm correlation coefficient, value of the results was over 0.9 for both citric acid-modified and -unmodified straw. Moreover, a comparison of the Freundlich capacitive factors (*K*_f_) clearly demonstrates the increased adsorption process efficiency as a result of citric acid modification of the straw. In turn, the energetic heterogeneity of the surface, determined by the Freundlich exponential coefficient, was significantly higher for straw modified by the citric acid, which may be indicated by the higher development of biosorbent surface due to etching with citric acid.

In the course of studies, additional analyses were conducted to elucidate the mechanism of biosorption process by analyzing of magnesium, calcium, and sodium ions concentrations in the reaction mixture at the adsorption equilibrium. Before the proper analysis of mentioned ion concentration at the adsorption equilibrium, there were also conducted additional analyses to determine the possible content of these ions in distilled water both before and immediately after the addition of straw to the initial solution. In all cases, the content of magnesium, potassium, and sodium ions did not exceed the AAS detection limits. Final results obtained for the mixtures at the adsorption equilibrium are shown in the table below (Table 1).

**Table 1:** Concentration of individual metals at equilibrium (mg/dm^3^) for modified and unmodified straw, including a standard deviation for each of the results

	Mg^2+^ (± SD)	Ca^2+^ (± SD)	Na^+^ (± SD)
Unmodified straw	4.5 ± 0.17	22.8 ± 3.26	1.2 ± 0.19
Straw after citric acid modification	0.7 ± 0.09	3.2 ± 0.48	122.6 ± 18.21
Straw after methanol modification	0.8 ± 0.17	4.7 ± 0.33	3.2 ± 0.71

Data presented in the table clearly indicate the relationship between the increase of the sum of the metal ions that were not present in the initial solution and the degree of zinc removal from the solution, for the solution with the initial zinc concentration equals to 80 mg/dm^3^ and mass:volume ratio of 1:200 cm^3^. This fact seems to indicate clearly on the nature of the biosorption as an ion-exchange process. It would also explain the decrease of efficiency in the case of straw modified with methanol, due to removing of calcium and magnesium ions from the surface of biosorbent.

Moreover, in order to verify the statistical significance of the results obtained during the study, a one-way ANOVA test was conducted to confirm if the used modifications actually affected to the observed biosorption properties of the straw. The tables below provide a detailed summary of the data for the ANOVA analysis, for each of the process conditions and straw modification types, both for the adsorption kinetic studies (Table 2) and adsorption isotherm analysis (Table 3).

**Table 2:** Results of one-way ANOVA analysis for adsorption kinetic studies including *F* statistic, *p* value, sum of squares (SS), and mean square (MS) for each of the measurement time

Adsorption time	3 min	10 min	20 min	40 min	70 min	120 min
*F* value	20.2373	47.6490	53.25794	40.90119	76.19559	57.12780
*p* value	0.00215	0.00021	0.00015	0.00032	0.00005	0.00012
SS	13.7860	16.5541	21.35165	20.84801	24.59895	22.71146
MS	6.89300	8.27708	10.67582	10.42400	12.29947	11.35573

**Table 3:** Results of one-way ANOVA analysis for adsorption isotherm studies including *F* statistic, *p* value, sum of squares (SS), and mean square (MS) for each of the biosorbent:solution ratio

Straw:solution ratio	1 g/200 cm^3^	0.8 g/200 cm^3^	0.6 g/200 cm^3^	0.4 g/200 cm^3^	0.2 g/200 cm^3^	0.1 g/200 cm^3^
*F* value	263.8	174.3	218.3	308.3	382.3	95.4
*p* value	0.000002	0.000005	0.000002	0.0000009	0.0000005	0.000028
SS	134.81	179.88	148.51	787.53	1276.47	708.54
MS	67.40	89.94	74.26	393.76	638.23	354.27

In order to compare the obtained values of the *F* statistics test with the tabular values, the correct number of degrees of freedom was determined; for this purpose, it was assumed a *k* value is equal to 3 (three types of straw were analyzed) and a sample size *N* is equal to 9. Thus, the values in the tables were compared with the *F* statistics *F*_(k,N-k)_, which in this case was *F* = 5.143 (Deniz and Karabulut 2017; Gonçalves et al. 2016). As it can be seen, all of the *F* statistics presented in Table 2, for each of the analyzed processes, significantly exceeded the critical *F*_(2.6)_ value, for a significance level alpha = 0.05. Therefore, it should be assumed that, with a given significance level, all results should be considered as statistically significant.

For the isotherm adsorption analysis, presented in Table 3, the situation was very similar to that for the adsorption kinetics. Once again, it was found that all of the obtained *F* statistics significantly exceeded the critical *F* (Deniz and Karabulut 2017; Gonçalves et al. 2016) value for a given significance level. That is why it can be assumed that all of the mentioned and presented results should be regarded as statistically significant, and chemical modifications used during the study had a significant effect on the change in the adsorption properties of the analyzed straw.

## Conclusions

The results obtained during the experiment showed that the biosorbent modification effect is strongly dependent on both of the adsorbent type and the substance that is removing in the adsorption process. The results differ from the literature data concerning the influence of biosorbents modification in the process of organic compound adsorption that seems to confirm the thesis of a strong specificity of the effectiveness of various modification methods depending on the type of removed pollutants. The type of chemical modification has an impact not only on the maximum adsorption capacity of biosorbents but also on the mechanism of the adsorption process. This fact is confirmed by the discrepancies between the assumptions of the Langmuir and Freundlich adsorption models and results obtained for modified and unmodified straw. Comparison of linear form of both types of adsorption isotherms shows that the result for modified biosorption process starts to meet the assumptions of the Langmuir adsorption theory, which again points to the emergence of changes in the structure of the straw surface. Conducted study showed also that despite numerous data and reports in the literature, the process of chemical modification of biosorbents is very specific for each of the sorbent and adsorption process. That is why results for even very similar conditions of the process may significantly differ from the literature predictions. That is why study of the properties and conditions of biosorbent chemical modification should be carried out taking into account the particular process conditions, type of biosorbent, and removal of heavy metals, every time before the main specific water purification process.

Another conclusion from the conducted study was that analyzed biosorption process was based on the ion-exchange adsorption mechanism. It was found on the basis of the presence of large amounts of calcium and magnesium ions in the final solution despite their absence in the initial solution. Analysis of magnesium and calcium ion concentrations showed also that after the modification of the straw by using a citric acid, they have been almost completely removed from the surface of the biosorbent. Moreover, as a result of the neutralization process, sodium ion concentration on the surface significantly increases, which in turn precludes clear determination whether the adsorption capacity of straw was increased due to the citric acid influence or rather as the effect of neutralization process by sodium hydroxide solution. Therefore, it seems that further studies are necessary to determine precisely the mechanism of proposed biosorbent modifications.
